# Role of the Medial Olivocochlear system among children with ADHD

**DOI:** 10.1590/S1808-86942012000300006

**Published:** 2015-10-14

**Authors:** Valéria Reis do Canto Pereira, Maria Ângela Guimarães Feitosa, Luiz Henrique Mourão do Canto Pereira, Marisa Frasson de Azevedo

**Affiliations:** a Ph.D. in Psychology (Behavior and Neurosciences) – University of São Paulo (Full Researcher/Collaborator at the Department of Basic Psychological Processes of the Psychology Institute – University of Brasilia).; b Ph.D. in Psychology (Psychobiology) - The University Of Michigan (Full Professor – Department of Basic Psychological Processes of the Psychology Institute – University of Brasília).; c Post-Doctorate – Medical School of the University of São Paulo General Coordinator of Biotechnology and Health – Ministry of Sciences and Technology).; d Ph.D. in Human Communications Disorders – Federal University of São Paulo (Associate Professor – Department of Speech and Hearing Therapy – Federal University of São Paulo).

**Keywords:** attention deficit disorder with hyperactivity, child, efferent pathways, otoacoustic emissions, spontaneous, supression

## Abstract

Attention deficit hyperactivity disorder (ADHD) patients show, as one of the main symptoms, an attentional impairment. Selective attention in the hearing process is the ability to understand speech in a noisy environment, which can be evaluated by several methods. One of the main approaches is the functioning of the Medial Olivocochlear Efferent System, which can be accessed by Transient-Evoked Otoacoustic Emissions (TOAE).

**Objective:**

This study aimed at evaluating the suppression effect of contralateral noise on TOAE in ADHD (study group) and normal subjects (control group). Study Design: Case-control study.

**Material and Methods:**

A study with 20 children distributed in two, age- and gender-matched groups. Results: No differences were found in TOAE responses between the two groups, with and without noise.

**Conclusions:**

We conclude that there were no functional differences in the Medial Olivocochlear Efferent System in the two groups analyzed.

## INTRODUCTION

The Attention Deficit/Hyperactivity Disorder (ADHD) is described by the *American Psychiatric Association - APA*^1^ as “a persistent pattern of attention deficit and/or hyperactivity, more frequent than what is usually seen in the equivalent level of development”. According to the APA, attention deficit and hyperactivity/impulsiveness symptoms manifest in different degrees and, in order to be classified as ADHD, they must manifest before 7 years of age; besides impacting in social development and functioning, academic and occupational lives of individuals, the losses must be present in at least two contexts such as, for example, at home and at school. Still, according to the APA, the ADHD is more frequent in males and its prevalence is estimated to be between 3% and 5% in school aged-children.

Children with ADHD have attention deficit as one of their main symptoms. Attention plays a role in the selection of some stimuli, providing the better processing of them. As far as hearing is concerned, selective attention is the ability to understand speech in noisy environments, and it can be assessed in different ways – one of them is the workings of the Medial Olivocochlear System Efferent.

The Medial Olivocochlear System Efferent has been studied especially because of its role in selective attention. This system can be assessed by the Otoacoustic Emissions Test, which involves low intensity sounds captured in the external acoustic meatus, associated with the mobility and mechanical ability of the external hair cells, apparently managed by the efferent pathways of the olivocochlear system. Otoacoustic emissions may be evoked by transient stimuli (click) which, because of having a broad array of frequencies, stimulates the entire cochlea (TEOAE).

Contralateral noise affects the transient evoked otoacoustic emissions (TEOAE) and it activates the efferent paths of the olivocochlear emissions, impacting the cochlear process responsible for the generation of the TEOAE, which represents suppression. This suppression effect was noticed by numerous authors such as the reduction in the response level of spontaneous and evoked otoacoustic emissions with the use of contralateral acoustic stimulation^2^, ^3^.

Ryan et al.^3^ reported that contralateral acoustic stimulation reduces TEOAE amplitude. This phenomenon may be mediated by the medial olivocochlear efferent system and, thus, its presence may be used to assess the integrity of the neural communication from one cochlea to another.

Some functions of the medial olivocochlear efferent system involve its role in the efficiency of signal capture in the presence of simultaneous noise, in the protection against lesions caused by high noise, in the control of the cochlear mechanical status and in auditory attention^4^.

Pillsbury et al.^5^ suggested that a reduction in auditory processing test performance involving the binaural process assessment may be a sensitive indicator of an “attention disorder” in patients with ADHD. The authors state that children with ADHD have a bad performance in the presence of the signal, because of a failure at this stage in the auditory processing.

Since there is a possibility of the medial olivocochlear efferent system be related to the ability of selective attention there was an interest in studying the function of such system in children diagnosed with ADHD. Moreover, there is a scarcity of papers associating this suppression effect in ADHD patients.

The present study aimed at comparing, by means of TEOAE, the suppression effect of the medial olivocochlear efferent system by contralateral auditory stimulation in normal children and in those with ADHD.

## MATERIALS AND METHODS

We recruited 30 children, of whom 20 participated in the study. They were divided in two groups, one made up of 10 children with ADHD and another made up of 10 children without ADHD (Control). The study was of the case-control type. The two groups were paired by gender and age. Each group was made up by three female and seven male children. The mean age of the children in the group was 9.8 years (Standard Deviation (SD) = 1.8 years) and that of the control group was 9.7 years (SD = 1.9 years). The study was carried out in the university clinic, after its approval by the Ethics in Research Committee of the Institution (Protocol # 0398/03) and the Informed Consent Form was signed by the guardians of the children from both groups.

As main complaints, the participants had difficulties in paying attention and, after having been diagnosed by a pediatric neurologist as having ADHD using the diagnostic criteria of DSM IV, they were referred for assessment by TEOAE. The group inclusion criteria were: otoscopy, immittance, normal bilateral tonal audiometry and the presence of TEOAE. The inclusion criteria for the ADHD group were the same inclusion criteria used for the Control group; however, its participants had a diagnosis of ADHD without the use of medication to treat the disorder. Exclusion criteria for both groups were: changes found in otoscopy, immittance measures, tonal audiometry and no TEOAE.

TEOAE measures were carried out using the ILO 96 cochlear emissions analyzer, from OTODYNAMICS, version 5.60, with the help of a probe fit to the external acoustic meatus of the subject, in an acoustic booth with sound insulation. We used a non-linear click stimulus in dBSPL, with regular 80 µs pulses, of rarefaction polarity, at a frequency of 50 Hertz (Hz). The stimulus intensity was 75 to 83 dBSPL, in sync with the procedures used by other authors^6^, ^7^. For the contralateral acoustic stimulation, we used the continuous white noise transmitted by means of a MAICO 17, ANSI-69 standard audiometer, through a TDH-39 phone at 50 dBSPL. The phone was coupled to the ear contralateral to the otoacoustic emissions prior to beginning the measuring, thus avoiding manipulating the probe between the different phases of assessment.

The response acceptance criteria were chosen according to the criteria adopted in the current literature, and were 70% higher in percentage of reproducibility values^6^, ^8^ and test tone probe setting stability higher than 70%^9^, representing a comparison of the level of stimulation recorded in the external acoustic meatus from test onset all the way to its conclusion. The number of stimuli utilized during TEOAE recording did not vary (260 series of four clicks), following the order of recording TEOAE in the right ear without and with a contralateral white noise; and with and without a contralateral noise for the left ear.

We used TEOAE, in the absence and presence of contralateral white noise, in both ears of all individuals in the sample. The responses were considered present when the signal/noise ratio was higher than 3 dB in the frequencies of 1000 Hz, 1500 Hz, 2000 Hz, 3000 Hz and 4000 Hz. In order to observe the suppression effect, we recorded the TEOAE with and without noise and we assessed the occurrence of changes in the TEOAE response level. We considered the presence of the suppression effect when there was a reduction greater than or equal to 1 dB from the response level with contralateral white noise.

For statistical purposes, we used the Wilcoxon, Mann Whitney Y and Equality of Two Proportions tests, adopting a significance level of 0.05%.

## RESULTS

In order to better understand the results obtained, their descriptions were organized in two parts, first checking the ear effect and, following, the group effect and stimulation settings.

### Comparing the Transient Evoked Otoacoustic Emissions in the Study and Control groups in relation to the Ear variable

The result shows the comparison between the Right (RE) and Left (LE) ears for the study (Table 1) and control (Table 2) groups in relation to amplitude and TEOAE differences with and without noise. There were no statistically significant differences between the groups.

**Table 1:** TEOAE response amplitude (in dBSPL) without and with contralateral noise and differences obtained without and with noise in the study group.

Study Group		Mean	SD	N	*p*
Without Noise	RE	12.05	3.73	10	0.742
	LE	11.50	3.62	10	

With Noise	RE	10.62	3.98	10	0.986
	LE	10.59	3.44	10	

Difference	RE	1.43	1.23	10	0.388
	LE	0.91	1.39	10

**Table 2:** TEOAE response amplitude (in dBSPL) without and with contralateral noise and differences obtained without and with noise, in the Control group.

Control Group		Mean	SD	N	*p*
W/out Noise	RE	10.85	2.64	10	0.701
	LE	10.30	3.60	10	

With Noise	RE	9.30	3.03	10	0.685
	LE	8.74	3.04	10	

Difference	RE	1.55	1.05	10	0.983
	LE	1.56	1.06	10	

Since we found no statistically significant difference between the ears in relation to the variables, the analysis started to be done together for both ears.

### Comparing the Transient Evoked Otoacoustic Emissions between the Study and Control groups

At this stage, we tried to see whether there was any difference in TEOAE responses without and with noise between the children of the control and those of the study group. There were no statistically significant differences between the groups. The results from this analysis are depicted on Table 3.

**Table 3:** TEOAE amplitudes without and with noise in relation to the Group variable.

Groups	Situation	Mean	SD	N	*p*
Study	w/out noise	11.78	3.59	20	0.264
Control	w/out noise	10.58	3.08	20	
Study	With noise	10.61	3.62	20	0.138
Control	With noise	9.02	2.97	20	

Following that, we tried to see if TEOAE differences without and with noise were different between the Study and Control groups. There were statistically significant differences between the groups. The results from this analysis are depicted on Table 4.

**Table 4:** Differences between TEOAE without and with noise (in dB) in relation to the Group variable.

Groups	Mean	SD	N	*p*
Study	1.17	1.31	20	0.307
Control	1.56	1.03	20	

We tried to investigate the quantification of the suppression effect for the right ear, left ear, and for both ears, from the study and control groups. There was no statistically significant difference between the groups. The results from these analyses are presented on Table 5.

**Table 5:** Suppression effect (in DB) in the Study and Control groups for the Right Ear (RE), Left Ear (LE) and both ears (RE+LE).

Groups	RE			LE			RE + LE		
	< 1dB	1 a 1.5dB	> 1.5dB	< 1dB	1 a 1.5dB	> 1.5dB	< 1dB	1 a 1.5dB	> 1.5dB
Study	n=2	n=1	n=7	n=4	n=1	n=5	n=6	n=2	n=12
Control	n=1	n=3	n=6	n=2	n=1	n=7	n=3	n=4	n=13
*P*	0.531	0.264	0.639	0.329	1.000	0.631	0.256	0.376	0.744

## DISCUSSION

The present study included ten children with ADHD - seven (70%) were boys, pointing to a greater prevalence of this disorder among males^1^, ^10^, ^11^, ^12^.

Initially, we tried to check whether the TEOAE without and with noise and the differences obtained without and with noise varied in relation to ear side (RE x LE), in both groups. There was no statistically significant difference in the Study group (Table 1) or in the Control group (Table 2). After having these results, the data from the two ears were combined, according to Burguetti & Carvallo^13^.

We investigated if the TEOAE amplitudes without and with noise varied between the two groups (Table 3). The results showed that there were no statistically significant differences between the groups. In both groups, the responses with noise were lower than those without noise, corroborating the findings from numerous authors^3^, ^14^, ^15^, ^16^.

Following that, we noticed that the values of the differences obtained without and with noise varied between the groups. There were no statistically significant differences between the groups (Table 4), corroborating other studies carried out^17^, ^18^.

The quantification of the suppression effect was studied for both ears separately. The statistical analysis carried out for the Right Ear (RE), Left Ear (LE) and both ears (RE+LE) did not show statistically significant differences between the groups studied (Table 5). Although it was not the objective of the present study to compare the suppression effect between RE and LE, the studies from Khalfa & Collet^19^ and Fávero^20^ showed that there is a greater activity of the right side efferent system; however, Rabinovich^21^ noticed a significant statistically significant difference for the LE.

According to the results fond in our study, they suggest there were no significant difference in the workings of the medial olivocochlear efferent system in the group with ADHD children, when compared to children of the same age and without such diagnosis. With our findings we can infer that the priority attentional involvement in ADHD patients are not of auditory sensorial mode; however, in order to confirm this hypothesis it would be important to broaden the number of cases studied.

We may also question whether auditory attention complaints in ADHD patients are associated with the existence of comorbid disorders, such as, for example, Central Auditory Processing Disorder, having that in many patients with ADHD this comorbidity was present^22^, ^23^.

Current literature shows a straight relationship of the superior olivary complex with the ability of sound location in space^24^. Based on this fact and on the data found in our study, we suggest the investigation of the olivocochlear efferent system by means of other measures, such as those of behavioral nature, in order to check for occasional difficulties in the auditory perception of patients with ADHD.

We did the present study starting from the principle that one of the ways used to assess Auditory Selective Attention happens through the analysis of the Medial Olivocochlear efferent system and that children with ADHD had important loss in attention.

As we studied the functioning of this system in ADHD children and in normal children, we could conclude that the TEOAE and the differences obtained without and with noise were similar in both groups studied. Thus, it has been suggested that there are no differences in the functioning of the medial olivocochlear efferent system in the children with attention deficit hyperactivity disorder, when compared to normal children; however, there is a need to go more in depth in this hypothesis by means of other methods capable of assessing the auditory function of ADHD patients such as, for example, the use of behavioral measures.

## CONCLUSIONS

The suppression effect which assesses the functioning of the medial olivocochlear efferent system by TEOAE with and without noise in children with ADHD did not show any difference when compared to the Control group in the present study. We did not find evidence, in this sample, that the medial olivocochlear efferent system is impacted in the presence of ADHD.
